# Lignocellulose-derived thin stillage composition and efficient biological treatment with a high-rate hybrid anaerobic bioreactor system

**DOI:** 10.1186/s13068-016-0532-z

**Published:** 2016-06-06

**Authors:** Margreet J. Oosterkamp, Celia Méndez-García, Chang-H. Kim, Stefan Bauer, Ana B. Ibáñez, Sabrina Zimmerman, Pei-Ying Hong, Isaac K. Cann, Roderick I. Mackie

**Affiliations:** Institute for Genomic Biology, and Department of Animal Sciences, Energy Biosciences Institute, University of Illinois at Urbana-Champaign, 1207 W Gregory Dr, Urbana, IL 61801 USA; Department of Animal, Life and Environment Science, Biogas Research Center, Hankyong National University, 327 Jungang-ro, Anseong-si, Gyeonggi-do 456-749 South Korea; Energy Biosciences Institute, University of California at Berkeley, 120A Energy Biosciences Building, 2151 Berkeley Way, MC 5230, Berkeley, CA 94729, USA; BP Biofuels, University of California at Berkeley, 120A Energy Biosciences Building, 2151 Berkeley Way, MC 5230, Berkeley, CA 94729 USA; Biological and Environmental Sciences and Engineering Division (BESE), Water Desalination and Reuse Center (WDRC), King Abdullah University of Science and Technology (KAUST), Thuwal, 23955-6900 Saudi Arabia

**Keywords:** Thin stillage, Lignocellulose, Methane, Anaerobic digestion, Mesophilic, Thermophilic, Hybrid reactor

## Abstract

**Background:**

This study aims to chemically characterize thin stillage derived from lignocellulosic biomass distillation residues in terms of organic strength, nutrient, and mineral content. The feasibility of performing anaerobic digestion on these stillages at mesophilic (40 °C) and thermophilic (55 °C) temperatures to produce methane was demonstrated. The microbial communities involved were further characterized.

**Results:**

Energy and sugar cane stillage have a high chemical oxygen demand (COD of 43 and 30 g/L, respectively) and low pH (pH 4.3). Furthermore, the acetate concentration in sugar cane stillage was high (45 mM) but was not detected in energy cane stillage. There was also a high amount of lactate in both types of stillage (35–37 mM). The amount of sugars was 200 times higher in energy cane stillage compared to sugar cane stillage. Although there was a high concentration of sulfate (18 and 23 mM in sugar and energy cane stillage, respectively), both thin stillages were efficiently digested anaerobically with high COD removal under mesophilic and thermophilic temperature conditions and with an organic loading rate of 15–21 g COD/L/d. The methane production rate was 0.2 L/g COD, with a methane percentage of 60 and 64, and 92 and 94 % soluble COD removed, respectively, by the mesophilic and thermophilic reactors. Although both treatment processes were equally efficient, there were different microbial communities involved possibly arising from the differences in the composition of energy cane and sugar cane stillage. There was more acetic acid in sugar cane stillage which may have promoted the occurrence of aceticlastic methanogens to perform a direct conversion of acetate to methane in reactors treating sugar cane stillage.

**Conclusions:**

Results showed that thin stillage contains easily degradable compounds suitable for anaerobic digestion and that hybrid reactors can efficiently convert thin stillage to methane under mesophilic and thermophilic conditions. Furthermore, we found that optimal conditions for biological treatment of thin stillage were similar for both mesophilic and thermophilic reactors. Bar-coded pyrosequencing of the 16S rRNA gene identified different microbial communities in mesophilic and thermophilic reactors and these differences in the microbial communities could be linked to the composition of the thin stillage.

**Electronic supplementary material:**

The online version of this article (doi:10.1186/s13068-016-0532-z) contains supplementary material, which is available to authorized users.

## Background

In the United States, there are around 200 operating ethanol biorefinery plants almost all from corn grain as a feedstock. These are mainly located in the Northern and Central States of Iowa, Nebraska, Kansas, South Dakota, Minnesota, Illinois, and Indiana. The plants produced 58 % of the global ethanol production (14.3 billion gallons in 2014). Brazil also significantly contributes 25 % of world ethanol production. Other producers are in Europe (6 %), China (3 %), Canada (2 %), Argentina (1 %), India (1 %), Thailand (1 %), and the rest of the world (3 %) [1]. This ethanol is blended with a petroleum-based fuel, initially as 3–10 % ethanol mixtures, and more recently, also higher ethanol percentages are used, such as in E15 (15 % ethanol) and E85 (85 % ethanol) [1].

For every liter of bioethanol produced, up to 20 L of thin stillage may be generated [2]. Stillage can be concentrated to produce syrup that can be used as an animal feed supplement [3]. Conversion of thin stillage by anaerobic digestion is an interesting alternative as it reduces the amount of carbon in stillage that would otherwise exceed the permissible discharge limits by producing a renewable energy source in the form of methane. Digestion usually takes place in two temperature ranges, mesophilic (30–40 °C) and thermophilic (50–60 °C). In general, thermophilic conditions are preferred over mesophilic, because there is higher volatile solids reduction, faster biogas production rates and less microbial contamination [4], reduction of cooling costs when fermenting after steam pretreatment [5] and can facilitate downstream product recovery [6]. Methane generated from anaerobic digestion of thin stillage can, for example, be used for steam and electricity generation or as a substitute fuel to offset some of the energy requirements [2]. Application of anaerobic digestion to the effluent from a pilot-scale biorefinery using wheat straw as feedstock resulted in 30 % more carbon recovery than when only using bioethanol production [7].

Despite the advantage of performing anaerobic digestion, whole stillages typically contain a substantial amount of hemicellulose and lignin that, together with cellulose, form the plant cell wall complex, and these components may be resistant to biodegradation [8]. Furthermore, thin stillage, obtained after solids removal from whole stillage, still contains high amounts of solubles such as lactic acid, acetic acid and sodium that are toxic to yeast, rendering it unsuitable for recycling as anaerobic fermentation broth. The data on lignocellulosic stillage characteristics are extremely limited and highly variable, and should be better characterized as these parameters have a significant effect on the feasibility of performing anaerobic digestion on these stillages.

In this study, the physicochemical and elemental properties of thin stillage derived from the production of bioethanol from energy cane and sugar cane was determined. Furthermore, anaerobic digestion of thin stillage was conducted in three mesophilic (MHR) and three thermophilic hybrid reactors (THR) using thin stillage derived from energy cane and sugar cane as substrate. The study aimed to demonstrate the feasibility of performing anaerobic digestion of thin stillages to recover methane as an energy source, and the microbial communities that anaerobically ferment the energy cane and sugar cane stillages were further evaluated.

## Methods

### Thin stillage sampling and collection

Pretreatment, and fungal saccharification followed by yeast fermentation, was used to generate ethanol at the BP Advanced Biofuel Demonstration Plant (1.4 million gallon per year/40 ton per day capacity) located in Jennings, Louisiana. Three 55-gallon drums of energy cane thin stillage and three of sugar cane thin stillage were obtained that were representative of the whole single production runs from either energy cane or sugar cane according to the plant manager. After the drums were obtained, they were transported under refrigeration to the Department of Animal Sciences, UIUC, aliquoted in 5-gallon portions in around 35 sealed buckets (Homer bucket, Home Depot, USA) of energy cane stillage as well as of sugar cane stillage and stored at 4 °C.

### Liquid chromatography of thin stillage

Anions and organic acids were analyzed by hydroxide-selective anion exchange chromatography. Samples were injected onto a 250 × 4 mm AS11-HC column (Thermo Scientific) equipped with a 50 × 4 mm AG11-HC IonPac guard column kept at 30 °C. The instrument used was a Thermo Scientific IC-5000+ with cooled autosampler, isocratic pump, eluent generator, thermostatted column compartment, and suppressed conductivity detector. Compounds were eluted at a flow rate of 2 mL/min and a hydroxide gradient of 0.2 mM isocratic for 6 min, then over 5 min to 5 mM, then over 16 min to 40 mM. Detection was performed by suppressed conductivity.

Monosaccharides were analyzed by anion exchange chromatography using a Thermo Scientific IC-5000+ instrument as described above except that a pulsed amperometric detector was used. The column used was a 150 × 3 mm CarboPac PA20 (Thermo Scientific) equipped with a 30 × 3 mm CarboPac PA20 guard column (Thermo Scientific). Compounds were eluted isocratically with 2 mM hydroxide at a flow rate of 0.4 mL/min and detected by pulsed amperometric detection.

Glycerol, ethanol, 5-HMF, and furfural were analyzed using a 300 × 7.8 mm HPX-87H column (BioRad, Richmond, CA, USA) equipped with a 30 × 4.6 mm Micro-Guard Cation H cartridge (BioRad). Compounds were eluted isocratically with 5 mM sulfuric acid at a flow rate of 0.6 mL/min and detected by refractive index detection (glycerol, ethanol) or UV (5-HMF, furfural).

All compounds were quantified by external calibration using mixtures of standards (purity ≥98 %) or using the combined seven anion standard (Thermo Scientific, San Jose, CA, USA).

### Elemental analysis of thin stillage

Elemental analysis was performed using a Varian Vista Pro CCD simultaneous inductively coupled plasma optical emission spectrometer (radial torch configuration) with an SPS 3 autosampler (Varian, Palo Alto, CA, USA). Samples were nebulized for transport into the radio frequency ICP, where each of the elements emits a specific spectrum. Wavelength intensities were measured by the photosensitive CCD microchip, and data were computed and stored with the ICP Expert computer software (Varian). Particles in thin stillage were analyzed with a Costech 4010 elemental analyzer (Costech, Valencia, CA, USA). Acetanilide and apple leaves were used as standards. A combustion process using chromium oxide as a catalyst with masses separated using an internal GC column was used.

### Experimental setup and operation of hybrid reactors

Six 1.25-L laboratory scale hybrid bioreactors, based on a previous design [9], were used in this study. High-rate upflow recirculation of 300 mL/min in the UASB compartment is combined with an internal bioreactor filtration support carrying a packed bed of around 80 biofiltration rings (Siporax, Pentair Aquatic Eco-Systems, Inc, Apopka, FL, USA) to further improve anaerobic digestion. The warm water flow in the water jacket was set to 325 mL/min. The temperature of the water bath connected to the water jacket was set to 55 °C for MHR and to 70 °C for THR resulting in internal reactor temperatures of 40 and 55 °C, respectively. Reactors were fed continuously with thin stillage using a flow of 0.06, 0.36, 0.54 or 0.9 mL/min. This gave hydraulic retention times of 15, 2, 1.5, and 1 day(s), respectively. Chemical oxygen demand (COD) measures the oxidation of organic matter in a substrate and is an indirect measure of the amount of organic compounds present. The tCOD (total chemical oxygen demand in g/L) of the stillage was used to calculate the OLR (organic loading rate in g COD/L/d). Different OLRs were chosen to test bioreactor performance of hybrid reactors treating thin stillages derived from the bioethanol production with energy cane and sugar cane as source biomass. Reactors were run using individual independent schedules. Only periods in which the reactors were running with each OLR for a minimum of three turnovers were chosen, with the assumption that the three turnovers allowed a pseudo steady state in the reactor at that particular operating condition. This was also evaluated from the observation that the percentage of methane produced in biogas was within a 10 % range deviation. Only data obtained after the three turnovers were used for further analysis of bioreactor performance.

### Inoculum for the bioreactors

MHR and THR were seeded with sludge from different sources ensuring presence of mesophilic and thermophilic microbial communities capable of anaerobic digestion of thin stillage under the respective conditions. MHR1 and MHR2 were inoculated with material from stable mesophilic methane-producing communities from cattle manure (Sieber et al., manuscript in preparation) and MHR3 from a mixture of sludge derived from MHR1 and MHR2. THR1 was inoculated from a mixture of samples from five different thermophilic anaerobic digesters from the temperature-phased anaerobic digestion system of wastewater treatment plants, and THR2 and THR3 were seeded with a mixture from the same samples that THR1 was inoculated with plus supplemental sludge from THR1. Initially, the bioreactors were filled with thin stillage that was four times diluted (¼ stillage COD) and adjusted to pH 7, after at least three turnovers, this was changed to two times diluted stillage (½ stillage COD) and subsequently to full strength stillage.

### pH determination of bioreactor effluent

Effluent of the HR was used for analysis using an Accumet AB15 pH meter (Fisher Scientific, Pittsburgh, PA, USA) on a daily basis.

### Measurement of methane percentage in total gas and specific methane production of bioreactors

Analysis of methane concentration in the biogas was performed on a daily basis and by direct injection of 0.5 mL gas produced by the bioreactors in a gas chromatograph (Series 580 Thermal Conductivity Gas Chromatograph, Gow-Mac Instrument Co., Bethlehem, PA). The GC column was 183 cm × 6.4 mm o.d. packed with Porapak Q and the temperatures for the injection port, detector, and column were 80, 80, and 75 °C, respectively. Biogas production was monitored on a daily basis and measured by a Milli Gas Counter (MGC-10, Ritter, Bochum, Germany). Methane production per day was calculated from the total volume of gas produced (in mL), and the methane percentage was determined analytically. Subsequently, specific methane production was calculated using the soluble chemical oxygen demand (sCOD) that was used per day.

### COD analysis of thin stillage and bioreactor effluent

The organic strength of thin stillage and bioreactor effluent samples was determined using the COD2 Mercury-free reagent (Hach, Loveland, CO, USA) according to the manufacturers’ instructions. COD removal was based on the difference in COD of the feed and effluent.

### Statistical analysis

Differences of the concentration of thin stillage compounds were statistically tested using the students *t* test. Bioreactor performance results were used for statistical analysis with the stats package in the R environment using three-way ANOVA with the lm command [10].

### Pyrosequencing analysis of bacteria and archaea in the bioreactors

Effluent and biofilm (rings) samples from mesophilic and thermophilic hybrid reactors performing anaerobic digestion of energy cane or sugar cane stillage were used for DNA isolation (FastDNA SPIN kit for soil, MP Biomedicals, Irvine, CA) and following the manufacturer’s instructions. Both no input and clean ring samples were used as negative controls for the DNA isolation procedure. DNA integrity was checked on 1 % agarose gel, and DNA concentration was determined using NanoDrop (ND 2000, Thermo Fisher Scientific, Waltham, MA). DNA was subjected to PCR targeting the 16S rRNA gene and using 515F (GTGTGCCAGCMGCCGCGGT [11]) and 806R (GGACTACVSGGGTATCTAAT [12]) sequences to construct the primers generating a ~300 bp amplicon. The primers were assembled as follows: general forward primer = 454 Titanium Lib-L primer B/Library Key (TCAG)/515F, sample specific reverse primer = 454 Titanium Lib-L primer A/Library Key (TCAG)/12-base Multiplex Identifier/806R. PCR reactions were carried out using 1 × Phusion High Fidelity PCR Master Mix with HF buffer (New England Biolabs, Ipswich, MA), 0.4 µM of the forward and the reverse primers, 5 % DMSO, and 20 ng template DNA per reaction and reactions. The PCR program consisted of initial denaturation at 98 °C for 30 s, followed by 35 cycles of denaturation at 98 °C for 8 s, annealing at 60 °C for 25 s, and extension at 72 °C for 2 min with a final extension at 72 °C for 10 min after the last cycle. For PCR, reactions with no DNA template were used as negative control, and in these reactions, no visible PCR product was produced. DNA from a soil microbial community, which should have no or low archaeal relative abundance [13], was used as a control, and both the PCR negative reaction and DNA isolation negative samples were used as negative controls. PCR reactions were performed in triplicate, and each set of triplicates was combined and purified using the Zymo DNA clean and concentrator kit (Zymo Research, Irvine, CA) and quantified using the Qubit dsDNA BR assay kit (Life Technologies, Carlsbad, CA). These quantified samples were combined in equimolar ratios. Sample pools were quantified (Qubit dsDNA BR assay kit) and further processed at the Keck Center (University of Illinois at Urbana-Champaign, Urbana, IL). Sample pools were subjected to quality control including qPCR and quality check on a High Sensitivity DNA chip (Agilent, Santa Clara, CA). Subsequently, the pools were used for emulsion PCR using the Roche emPCR method (Roche Group, Basel, Switzerland). 454 pyrosequencing was performed using Roche GS FLX + system, v2.9, flow pattern A and analyzed through amplicon signal processing using Roche software version 2.9 (Roche Group, Basel, Switzerland).

### Analysis of the pyrosequencing data

The pyrosequencing data obtained, were analyzed using the QIIME pipeline [14]. We excluded reads with lengths below 200 bp and quality scores less than 25. No mismatches were allowed in the forward primer. The sequences were denoised and binned into operational taxonomic units (OTU) at a cut-off of 97 % similarity using uclust [15]. Cluster seed was used as representative sequence. Chimeric sequences were detected with Chimera Slayer and excluded [16]. Subsequently, the sequences were aligned with PyNAST using the Greengenes core set alignment as Ref. [17, 18]. Taxonomy was assigned by comparing to the database of the Ribosomal Database Project [19]. An OTU table was prepared, and phylogeny was constructed using RAxML [20]. Taxonomy results were plotted using Microsoft Excel. Further processing of the data involved between-sample diversity analysis, which compared the abundance and presence of microorganisms between the different samples. Based on the collated data, an unweighted UniFrac distance matrix was determined to compare the extent of similarities among samples, and the matrix was utilized for principal coordinate analysis on 3D biplots generated in Emperor [21].

## Results

### Organic substrates in thin stillage

The composition of thin stillage derived from energy cane (n = 3) and sugar cane (n = 3) was assessed for their suitability in anaerobic digestion. These stillages were obtained from a 40 ton/day cellulosic ethanol demonstration plant located in Jennings, Louisiana. The COD of both stillage types was relatively high, averaging at 43.4 and 30.1 g/L for energy cane and sugar cane stillage, respectively (Table 1), and compared to other types of thin stillage [22, 23]. Total and soluble COD were significantly different with around 31 % lower total COD in sugar cane stillage and around 36 % lower soluble COD in sugar cane stillage compared to energy cane stillage (Table 1). The total concentration of selected sugars (e.g., arabinose, galactose, glucose, xylose, mannose, and fructose) was over two hundred times higher in energy cane than in sugar cane stillage (Table 1). Acetic acid could not be detected in energy cane stillage, but was present in concentration of 45 mM in sugar cane stillage (Table 1). The lactic acid concentration in energy and sugar cane stillage was 37 and 35 mM, respectively (Table 1), and the succinic acid concentration was 1 mM in energy cane stillage and 0.5 mM in sugar cane stillage (Table 1). Other organic compounds were also found in thin stillage (Table 1). Glycerol was present at 10 mM in sugar cane but not in energy cane stillage. The high-energy compounds ethanol, 5-hydroxymethyl furfural, and furfural were found only in sugar cane stillage (Table 1).

**Table 1:** Physicochemical analysis of thin stillage from sugar and energy cane used for bioethanol production

Parameter	Energy cane stillage (*n* = 3)		Sugar cane stillage (*n* = 6)		*t* test
	(mg/L)^a^	(µM)	(mg/L)^a^	(µM)	*p* value
tCOD	43,398 ± 3133	NR	30,106 ± 2186	NR	0.0064 (#)
sCOD	40,279 ± .893	NR	25,875 ± 381	NR	0.00043 (#)
pH	4.30 ± 0.03	NR	4.32 ± 0.00	NR	0.051 (=)
C:N pellet^b^	11.2:1	NR	13.8:1	NR	NR
Ammonia	509.2 ± 26.5	9519.2 ± 496.3	260.5 ± 113.7	4869.5 ± 2126.0	0.00057 (#)
Arabinose	15.6 ± 0.7	104.1 ± 4.9	0.7 ± 0.1	4.8 ± 0.8	0.00065 (#)
Galactose	45.1 ± 0.0	250.3 ± 0.0	1.7 ± 0.3	9.6 ± 1.7	8.7 × 10^−12^ (#)
Glucose	1275.7 ± 7.2	7080.9 ± 39.7	0.4 ± 0.0	2.5 ± 0.2	1.1 × 10^−5^ (#)
Xylose	0.4 ± 0.7	2.8 ± 4.9	0.8 ± 0.3	5.5 ± 1.9	0.44 (=)
Mannose	1.4 ± 0.7	7.6 ± 3.8	0.3 ± 0.1	1.9 ± 0.6	0.12 (=)
Fructose	67.1 ± 16.1	372.6 ± 89.3	2.1 ± 0.9	11.6 ± 4.9	0.020 (#)
Succinic acid	169.6 ± 12.6	1436.1 ± 107.1	57.8 ± 2.3	489.3 ± 19.7	0.0038 (#)
Lactic acid	3376.1 ± 40.1	37,478.5 ± 445.5	3158.4 ± 27.3	35,062.6 ± 302.8	0.0040 (#)
Glycerol	NA	NR	937.2 ± 5.3	10,177.4 ± 57.8	–
Acetic acid	–	–	2710.2 ± 20.0	45,131.6 ± 332.9	–
Fumaric acid	<	<	–	–	–
HMF	NA	NR	6.8 ± 0.0	54.2 ± 0.4	–
Furfural	NA	NR	40.2 ± 0.5	417.9 ± 4.8	–
Ethanol	NA	NR	7290.0 ± 370.0	158,237.5 ± 8031.3	–
Fluoride	NA	NR	–	–	–
Chloride	206.4 ± 0.7	5822.3 ± 18.5	–	–	–
Nitrite	NA	NR	1.6 ± 2.2	34.5 ± 48.8	–
Bromide	NA	NR	0.4 ± 0.6	4.9 ± 7.0	–
Nitrate	NA	NR	0.6 ± 0.6	9.1 ± 8.9	–
Phosphate	216.1 ± 14.1	2275.5 ± 148.8	175.0 ± 57.8	1842.8 ± 608.6	0.13 (=)
Sulfate	2225.2 ± 4.6	23,162.6 ± 48.4	1743.9 ± 88.3	18,152.1 ± 919.1	1.5 × 10^−5^ (#)

^a^pH is expressed in units and C:N pellet is a ratio

^b^Single measurement of the small amount of pellet material obtained

*–* Not detected, < below detection limit, *NA* not analyzed, *NR* not relevant, # significantly different (*p* < 0.05), = not significantly different (*p* > 0.05)

### Chemical and physical properties of thin stillage

Both types of thin stillage had a pH of 4.3 (Table 1). In energy and sugar cane stillage, the ammonia concentration was 9.5 and 4.8 mM, respectively (Table 1). The sulfate concentration was 23.2 mM in energy cane and 18.2 mM in sugar cane stillage (Table 1). The chloride concentration was 5.8 mM in energy cane stillage but was not detected in sugar cane stillage (Table 1). Nitrite, nitrate, and bromide concentrations were less than 35 µM, and the phosphate concentration was 2.2 mM in energy cane and 1.8 mM in sugar cane stillage (Table 1). Thin stillage commonly contains high concentrations of phosphorus, potassium, and sulfur [22, 23]. In energy and sugar cane stillage, the concentration of these elements was 3.1 and 3.5, 5.2 and 2.4, 24.35 and 12.0 mM, respectively (Table 2). Also calcium and magnesium concentrations were high in energy cane and sugar cane stillage (16.4 and 9.4 plus 19.3 and 9.7, respectively, Table 2). Thin stillage contains particulates that contribute to 7.2 and 14.1 % of the total COD of energy cane and sugar cane stillage, respectively (Table 1). The C:N ratio of particulates obtained from energy cane and sugar cane stillage was 11.2:1 and 13.8:1, respectively (Table 1).

**Table 2:** Elemental composition of thin stillage from sugar and energy cane used for bioethanol production

Element	Name	Energy cane stillage (*n* = 3)		Sugar cane stillage (*n* = 6)		*t* test
		(mg/L)	(mM)	(mg/L)	(mM)	*p* value
Al	Aluminum	0.56 ± 0.02	0.02 ± 0.00	1.06 ± 0.15	0.04 ± 0.01	7.3 × 10^−6^ (#)
As	Arsenic	<	<	<	<	–
B	Boron	1.81 ± 0.35	0.17 ± 0.03	0.23 ± 0.03	0.02 ± 0.00	0.067 (=)
Ba	Barium	0.21 ± 0.01	0.00 ± 0.00	<	<	–
Be	Beryllium	<	<	<	<	–
Ca	Calcium	659.05 ± 5.80	16.44 ± 0.14	375.93 ± 2.83	9.38 ± 0.07	1.3 × 10^−12^ (#)
Cd	Cadmium	<	<	<	<	–
Co	Cobalt	<	<	0.04 ± 0.00	0.00 ± 0.00	–
Cr	Chromium	0.71 ± 0.01	0.01 ± 0.00	0.28 ± 0.00	0.01 ± 0.00	7.3 × 10^−11^ (#)
Cu	Copper	0.04 ± 0.01	0.00 ± 0.00	0.04 ± 0.01	0.00 ± 0.00	6.3 × 10^−5^ (#)
Fe	Iron	37.94 ± 0.35	0.68 ± 0.01	5.44 ± 0.05	0.10 ± 0.00	1.4 × 10^−6^ (#)
K	Potassium	201.53 ± 6.64	5.15 ± 0.17	93.46 ± 1.35	2.39 ± 0.03	1.1 × 10^−8^ (#)
Li	Lithium	<	<	<	<	–
Mg	Magnesium	470.01 ± 7.50	19.34 ± 0.31	234.96 ± 0.44	9.67 ± 0.02	1.9 × 10^−10^ (#)
Mn	Manganese	1.70 ± 0.05	0.03 ± 0.00	1.11 ± 0.00	0.02 ± 0.00	1.3 × 10^−6^ (#)
Mo	Molybdenum	<	<	0.08 ± 0.00	0.00 ± 0.00	–
Na	Sodium	3617.74 ± 73.67	157.36 ± 3.20	888.39 ± 9.32	38.64 ± 0.41	0.00066 (#)
Ni	Nickel	1.54 ± 0.04	0.03 ± 0.00	1.18 ± 0.00	0.02 ± 0.00	5.0 × 10^−9^ (#)
P	Phosphorus	97.23 ± 1.43	3.14 ± 0.05	109.07 ± 0.49	3.52 ± 0.02	1.9 × 10^−15^ (#)
Pb	Lead	<	<	<	<	–
S	Sulfur	780.79 ± 6.50	24.35 ± 0.20	384.71 ± 4.42	12.00 ± 0.14	5.2 × 10^−12^ (#)
Sb	Antimony	<	<	<	<	–
Se	Selenium	<	<	<	<	–
Si	Silicon	46.29 ± 12.79	1.65 ± 0.46	26.07 ± 0.44	0.93 ± 0.02	0.00027 (#)
Sn	Tin	<	<	<	<	–
Sr	Strontium	0.70 ± 0.01	0.01 ± 0.00	0.25 ± 0.00	0.00 ± 0.00	9.8 × 10^−9^ (#)
Ti	Titanium	0.03 ± 0.00	0.00 ± 0.00	0.02 ± 0.00	0.00 ± 0.00	1.3 × 10^−10^ (#)
Tl	Thallium	<	<	<	<	–
V	Vanadium	<	<	<	<	–
Zn	Zinc	1.71 ± 0.08	0.03 ± 0.00	0.78 ± 0.25	0.01 ± 0.00	0.0025 (#)

< Below detection limit, – not applicable, # significantly different (*p* < 0.05), = not significantly different (*p* > 0.05)

### Anaerobic fermentation of thin stillage to recover methane as an energy source

**Fig. 1:**
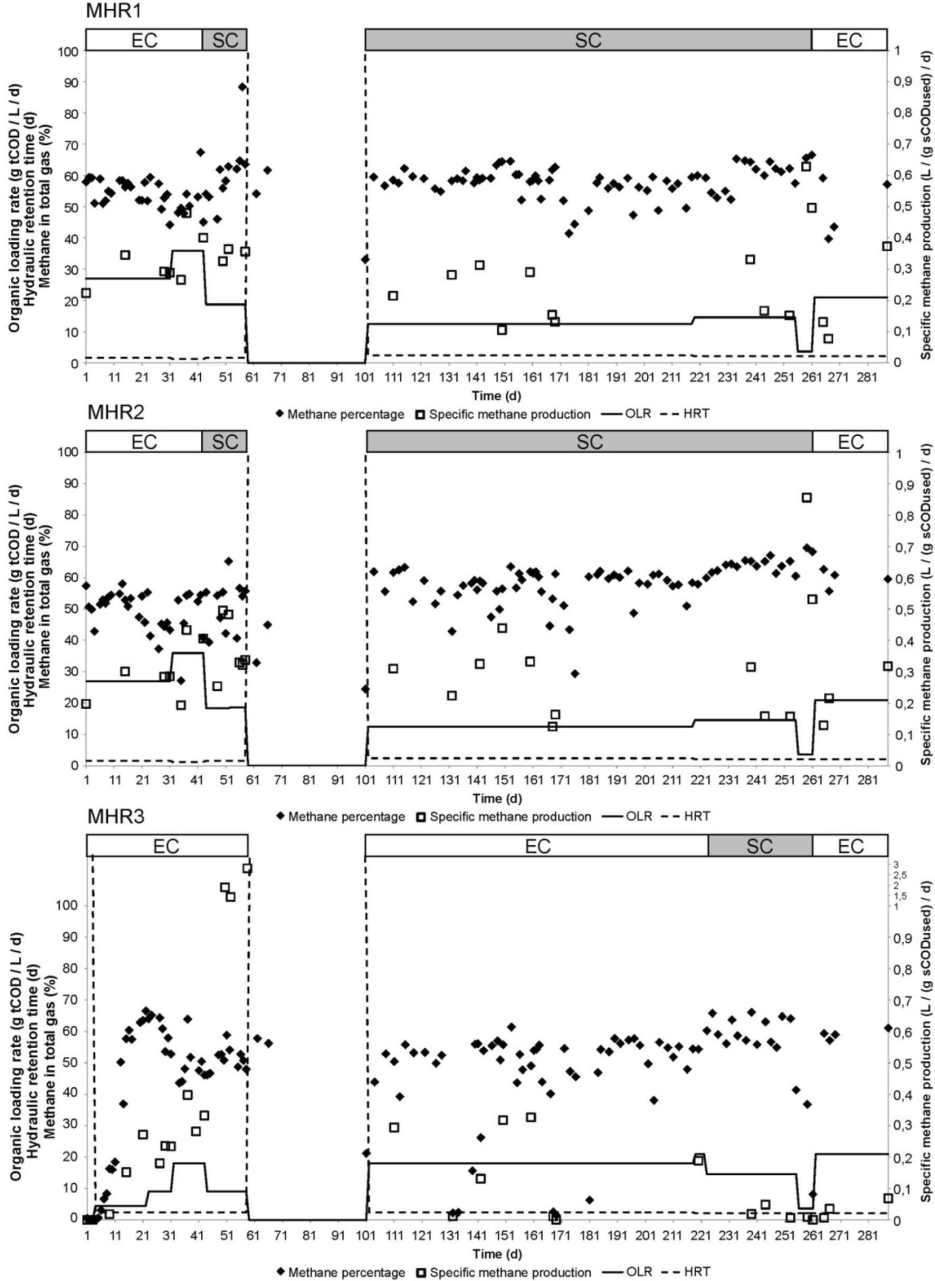
Methane percentage in biogas and specific methane production from anaerobic digestion of thin stillage. Energy cane stillage (EC) and sugar cane stillage (SC) were fed with the organic loading rate (OLR) and hydraulic retention time (HRT) shown in these time courses of the mesophilic hybrid reactors

**Fig. 2:**
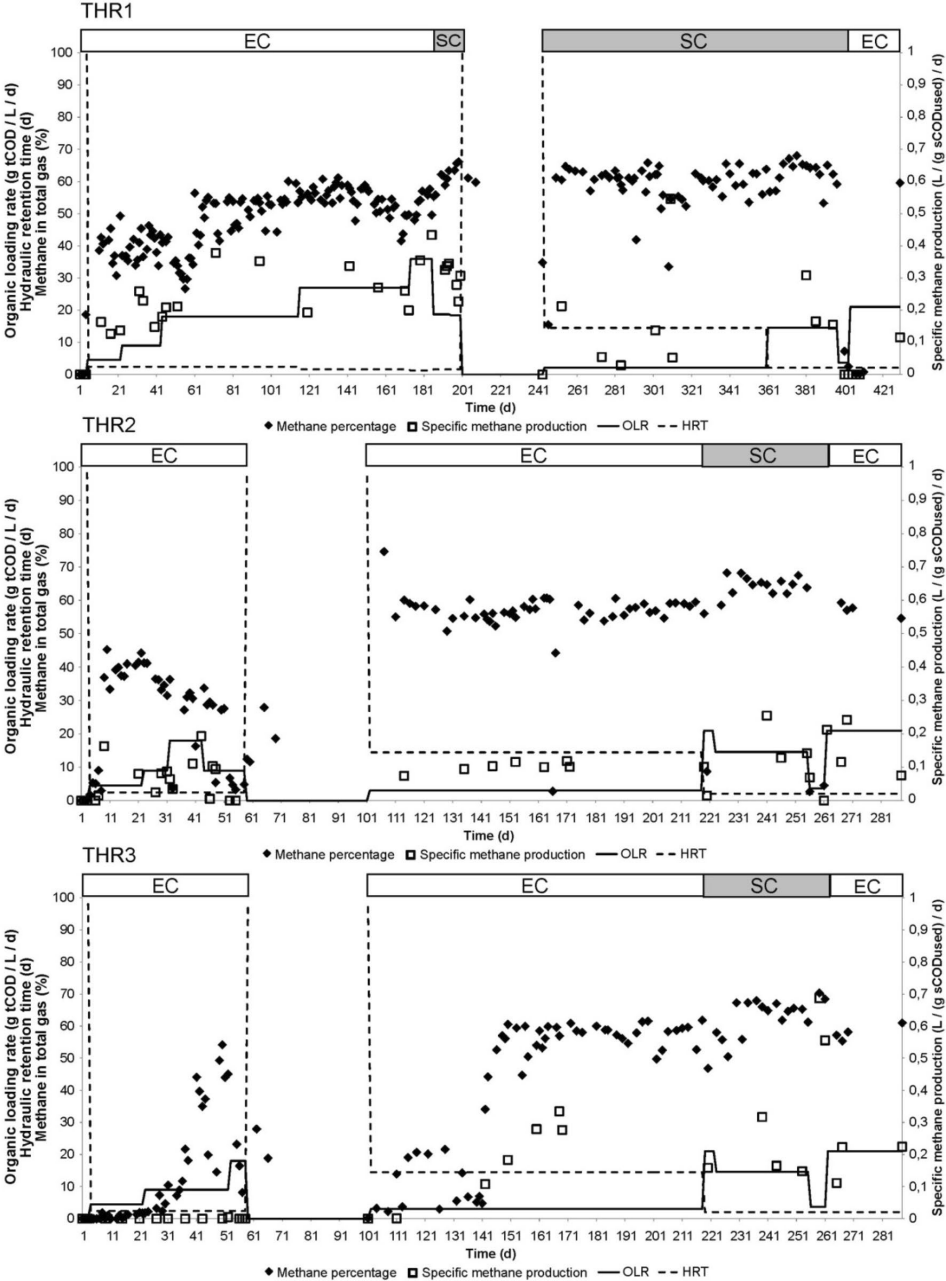
Methane percentage in biogas and specific methane production from anaerobic digestion of thin stillage. Energy cane stillage (EC) and sugar cane stillage (SC) were fed with the organic loading rate (OLR) and hydraulic retention time (HRT) shown in these time courses of the thermophilic hybrid reactors

Thin stillage was tested for its anaerobic digestibility in mesophilic and thermophilic hybrid reactor systems. This system was designed for efficient biogas production and combined upflow anaerobic sludge blanket and packed-bed technologies [9]. The methane percentage in the biogas produced was not very different under mesophilic and thermophilic conditions (Figs. 1, 2; Additional file 1: Figure S1). Methane percentage was decreased with decreased organic loading rate and became higher when the organic loading rate increased. Furthermore, the methane percentage was slightly higher when sugar cane rather than energy cane stillage was used (Figs. 1, 2; Additional file 1: Figure S1). In accordance with this, a three-way ANOVA (analysis of variance) showed that there was an effect of low or high organic loading rate (*p* = 0.002) and the type of stillage (*p* = 0.006) (Additional file 1: Table S1). Specific methane production increased with increasing organic loading rate, but with sugar cane stillage, very high production was reached with relatively low organic loading rate, which was not observed with energy cane stillage (Additional file 1: Figure S2). This was reflected in the three-way ANOVA that also showed that low or high organic loading rate had a significant effect on the methane production when taking main effects and interactions into account (*p* = 0.04), and there was also a significant interaction of low and high organic loading rate and the type of stillage (*p* = 0.03, Additional file 1: Table S1). This high methane production with low organic loading rate together with higher methane percentage in the biogas when sugar cane stillage was used, indicates that sugar cane stillage can be more efficiently converted to methane as compared to energy cane stillage.

### Anaerobic fermentation of thin stillage reduces COD of stillage discharge

**Fig. 3:**
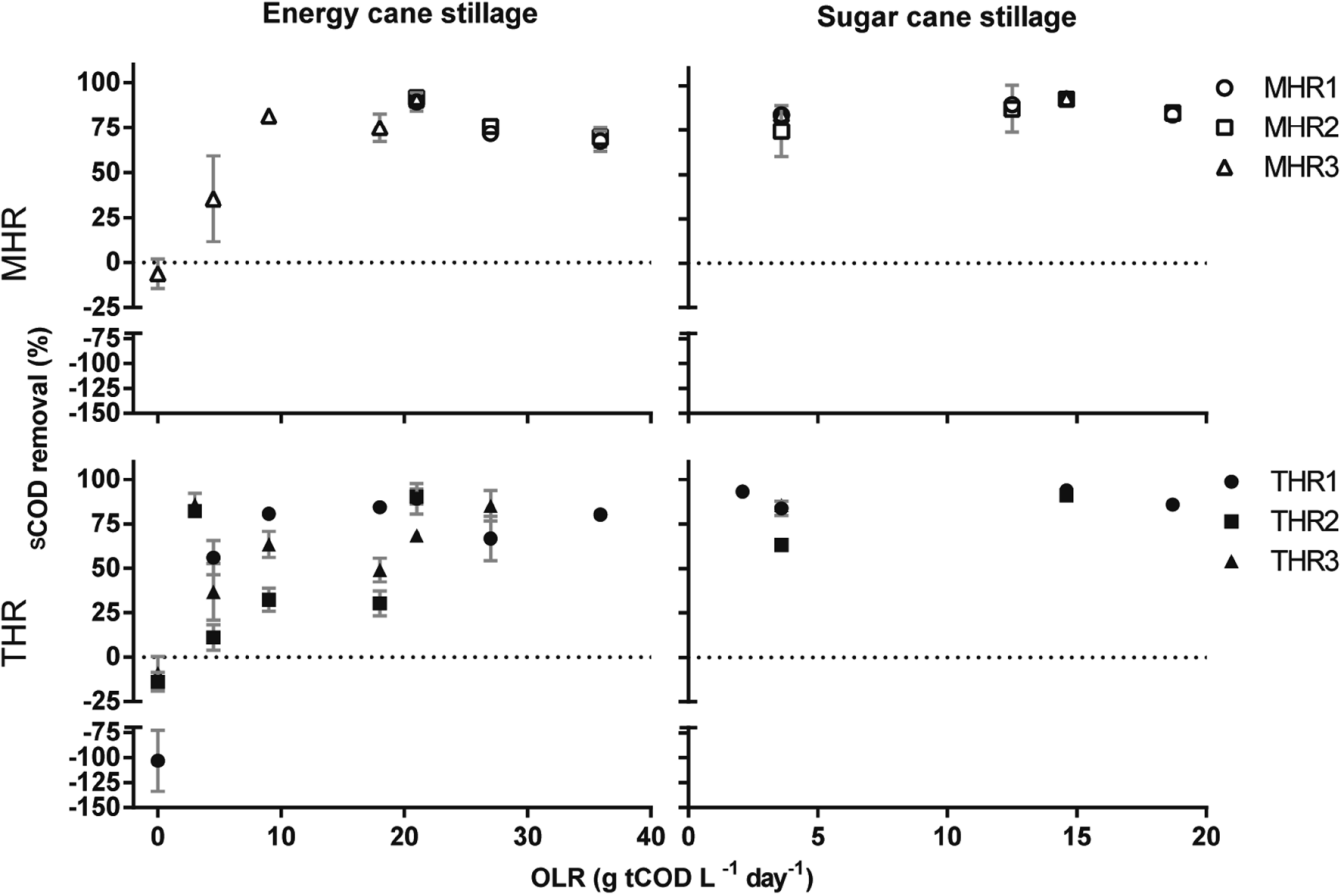
Soluble COD removal during anaerobic digestion of thin stillage. Effect of different organic loading rates of stillage derived from energy cane or sugar cane on hybrid reactor performance expressed as sCOD removal under mesophilic and thermophilic conditions. *Negative values* indicate higher effluent sCOD than influent sCOD in batch conditions

**Fig. 4:**
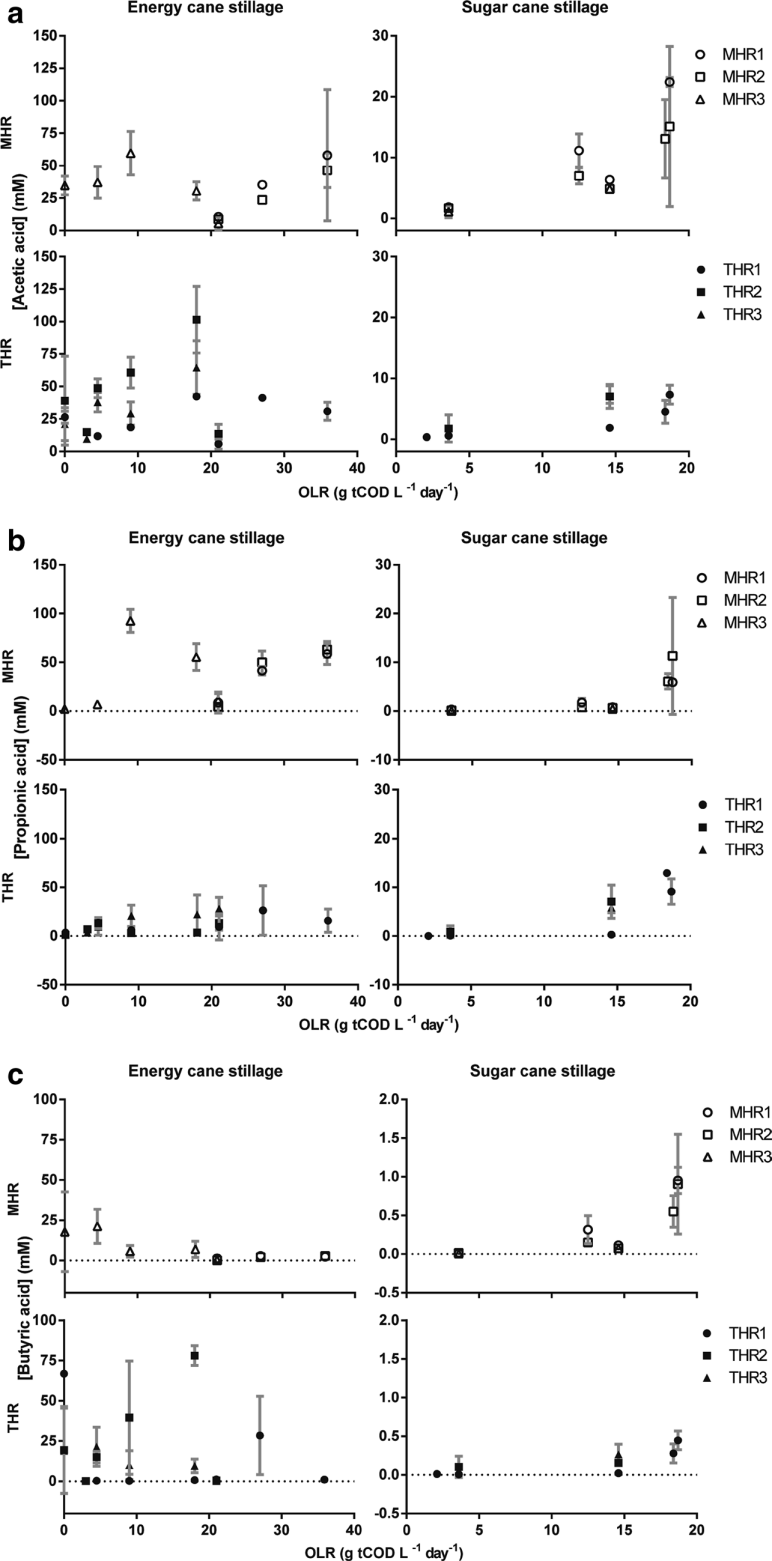
Volatile fatty acid concentration in effluent of hybrid reactors during anaerobic digestion of thin stillage. Effect of different organic loading rates of stillage derived from energy cane and sugar cane on the concentration of the volatile fatty acids **a** acetic acid, **b** propionic acid or **c** butyric acid under mesophilic and thermophilic conditions

The percentage of soluble COD from thin stillage that was removed by the hybrid reactors was determined. COD removal was up to 91.5 % in MHR and up to 90.5 % in THR using energy cane stillage and significantly higher using sugar cane stillage, up to 93.0 % in MHR and up to 93.8 % in THR (Fig. 3). ANOVA analysis with main effects and no interactions confirms that there was a significant effect of the type of stillage (p = 0.004) on COD removal (Additional file 1: Table S1). COD removal seems to increase until an organic loading rate of 15–21 g COD/L/d using both types of stillage and for both mesophilic and thermophilic temperature regimes, and experienced a slight decrease with higher organic loading rate (Fig. 3). Accordingly, ANOVA analysis with main effects and no interactions showed that there is a significant independent effect of low and high organic loading rate (p = 0.004) on COD removal (Additional file 1: Table S1). Effluent pH was also used as an indicator of bioreactor performance. Using energy cane stillage, the pH was between 6.45 and 7.54 for MHR and between 5.87 and 7.87 for THR, and using sugar cane stillage, the pH was between 7.13 and 7.36 for MHR and between 7.31 and 7.70 for THR (Additional file 1: Figure S3). When performing ANOVA using main effects and interactions, the difference between the two types of stillage was not significant, but a nearly significant *p* value of 0.06 was obtained for the effect of low compared to high organic loading rate. However, using only main effects, there was no significant difference between the low and high organic loading rate (*p* = 0.22, Table 3). MHR and THR have a high buffering capacity as the effluent pH is around neutral, and the feed is very acidic (Table 1). Also, the pH does not change drastically with different conditions (Additional file 1: Figure S3). Finally, VFA concentrations increased with increased organic loading rate, but this did not result in a decrease in the pH (Fig. 4; Additional file 1: Figure S3).

### Microbial community involved in biological treatment of thin stillage

**Fig. 5:**
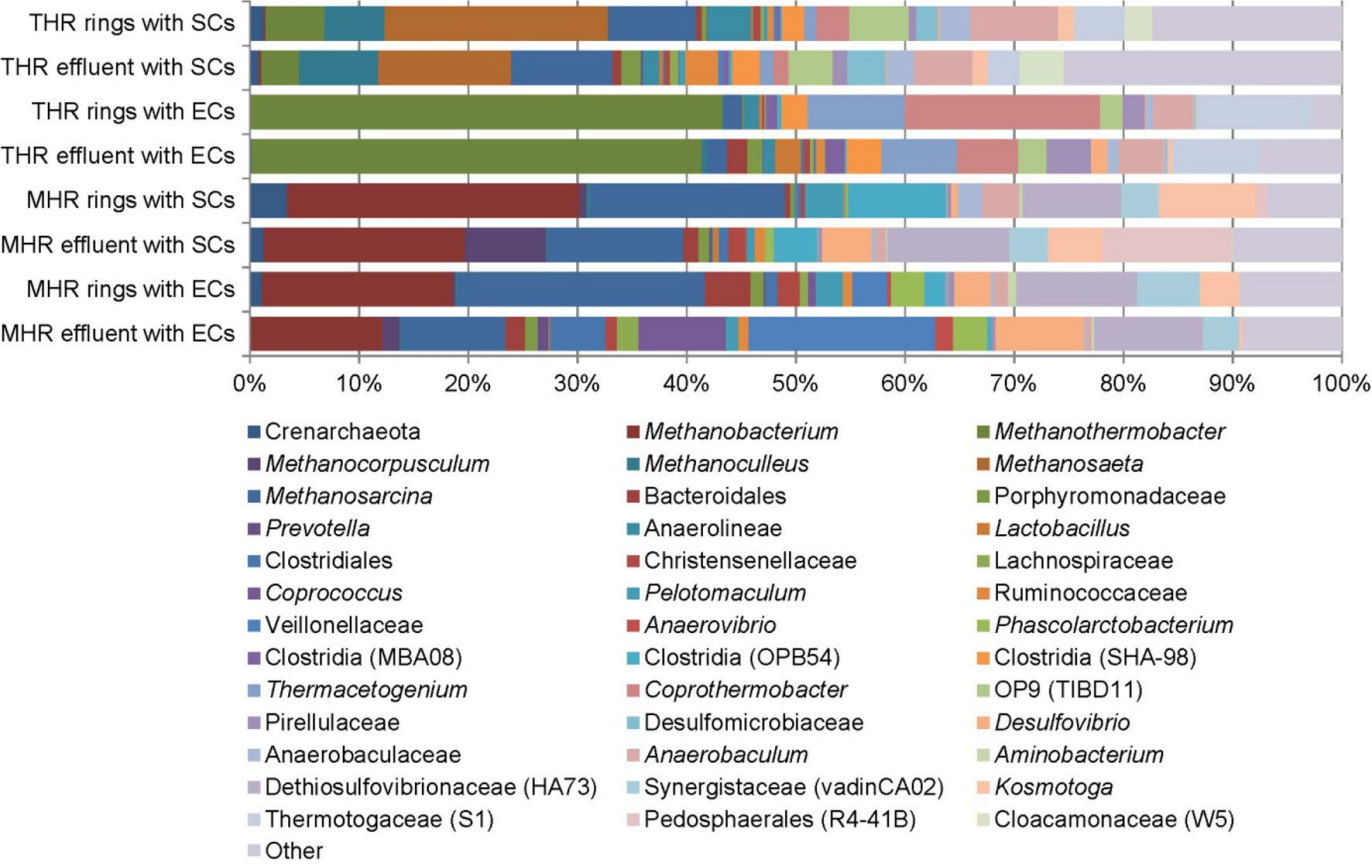
Microbial community composition of hybrid reactors performing anaerobic digestion of thin stillage. The effect of energy cane stillage (ECs) and sugar cane stillage (SCs) on microbial communities of rings and effluent in mesophilic (MHR) and thermophilic (THR) hybrid reactors

**Fig. 6:**
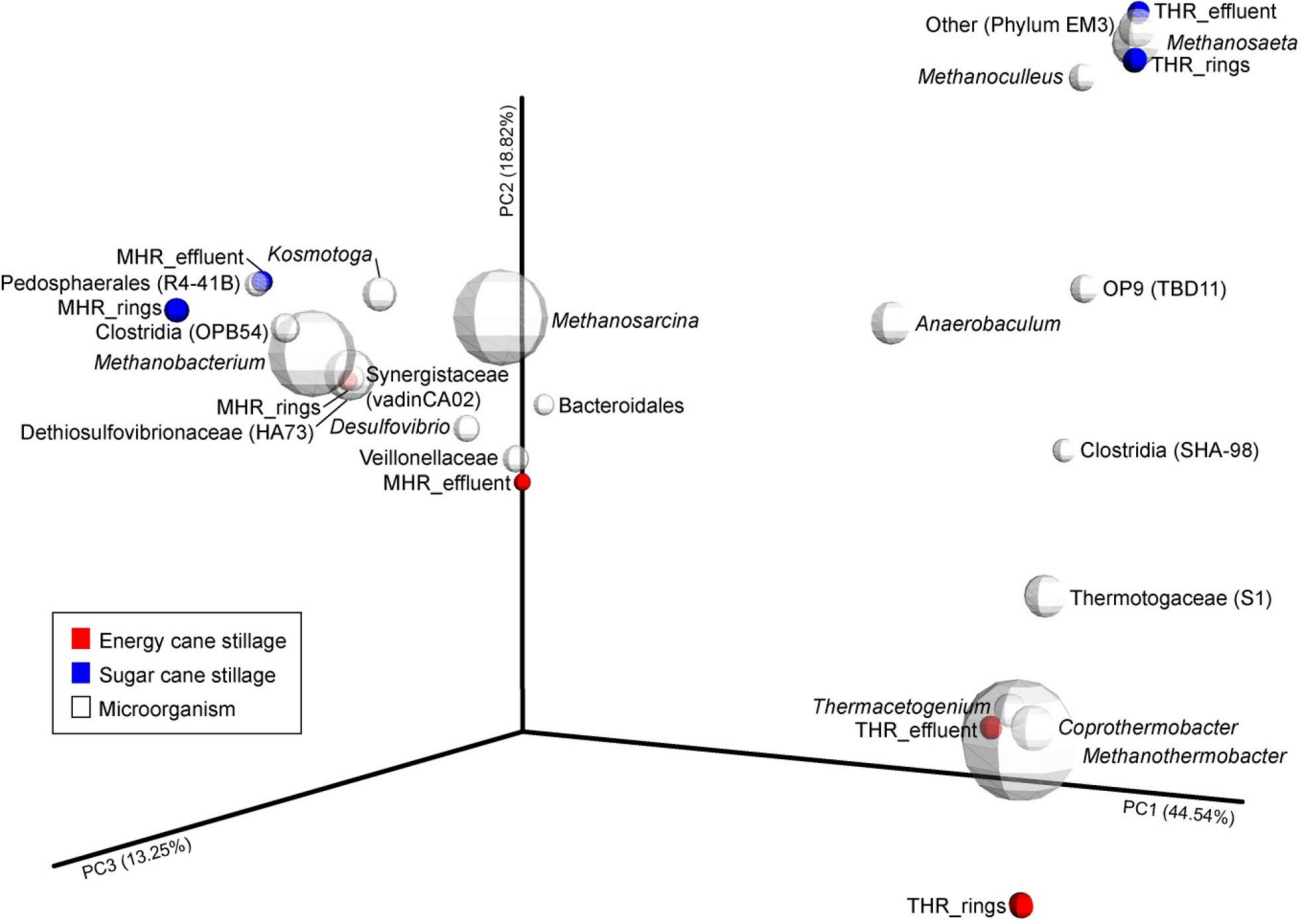
Between-sample diversity of microbial communities of effluent in rings in mesophilic and thermophilic hybrid reactors. The abundance (*sphere size*) and presence (*location*) of most abundant microbial families in these different communities are also shown. *Principal coordinates* (PC1, PC2, and PC3) are indicated along the axes

Microbial community analysis was performed to evaluate biological treatment of energy cane and sugar cane stillage in mesophilic and thermophilic hybrid reactors. Microbial community of the biofilm that attached to the ceramic rings of the packed bed was analyzed separately from the microorganisms present in the bioreactor effluent. Thin stillage was degraded by different microbial communities in mesophilic compared to thermophilic hybrid reactors. Furthermore, there were marked differences in microbial community composition of the reactors when treating energy cane stillage versus sugar cane stillage (Fig. 5). Between-sample diversity analysis compares the abundance and presence of microorganisms between the different samples. This also showed the abundance and presence of the most abundant species (Fig. 6). Energy cane stillage was treated by a thermophilic community in which *Methanothermobacter, Coprothermobacter*, and *Thermacetogenium* were more abundant. In contrast, *Methanosaeta, Methanoculleus*, and Phylum EM3 were more related to thermophilic sugar cane stillage treatment. For mesophilic treatment, Veillonellaceae, Synergistaceae, Dethiosulfovibrionaceae, and *Desulfovibrio* were more abundant in the anaerobic fermentation of energy cane stillage and Pedosphaerales to sugar cane stillage (Fig. 6). The between-sample diversity analysis also indicated that *Anaerobaculum* species and species belonging to OP9 (TBD11), the Clostridia (SHA-98) and Thermotogaceae (S1) were more related to thermophilic treatment. *Methanosarcina, Methanobacterium*, Bacteroidales, *Kosmotoga*, and Clostridia (OPB54) were more closely related to mesophilic treatment (Fig. 6).

## Discussion

The expansion and diversification of new alternative energy sources in a sustainable and efficient way is one of the major concerns of the industrialized world. Large-scale production of ethanol or other liquid transportation fuels from lignocellulosic biomass has the potential to replace a major portion of imported petroleum fuels and can potentially reduce greenhouse emissions of CO_2_. However, treatment and utilization of the resultant stillage is essential for lignocellulosic biomass to qualify as a source of green energy. In this study, we propose that it is feasible to use anaerobic digestion for additional energy recovery in the form of methane from the stillages, and for further reduction of thin stillage COD content that was found to otherwise exceed the permissible discharge limits.

However, some challenges remain to be resolved in utilizing stillage for anaerobic digestion and fermentation. To the best of our knowledge, stillage characterization for lignocellulosic feedstocks, particularly those originating from bioenergy crops, has not been comprehensively studied. A collation of existing data from the literature showed that depending on the pretreatment performed prior to ethanol fermentation, the chemical qualities of lignocellulosic stillage varied with the feedstock type [23] and would affect the stability of the anaerobic digestion process. In this study, it was determined that stillages from energy cane and sugar cane had low pH (pH 4.3, Table 1), likely due to the need for enzymatic, concentrated or dilute acid hydrolysis of the cellulosic feedstocks in the upstream processes [8, 24]. Optimal methane production occurs between pH range of 6.6 and 7.8, with an optimum at pH 6.8. A possible reduced production or failure of the anaerobic digestion may occur if the pH drops below 6.1 or increases above 8.3 [25]. Some hydrogenase enzymes producing dihydrogen that can be produced for methane production have reduced activity with low pH. For anaerobic digestion of solid waste, a C:N ratio between 20 and 35 is considered optimal [26]. The C:N ratio of pellets from energy cane and sugar cane thin stillage weas lower than the optimal ratio for anaerobic digestion. Hence, providing alkalinity, pH regulation, and additional N sources to maintain an optimal pH and C:N ratio would be necessary during anaerobic digestion of thin stillages. Other potential challenges that may hinder anaerobic digestion of the stillages include high sulfate concentrations, likely due to the use of dilute sulfuric acid in pretreatment of lignocellulosic biomass to expose cellulose and hemicellulose to enzymatic depolymerization [27]. Sulfate concentrations can promote the growth of sulfate-reducing bacteria which, in turn, outcompete hydrogenotrophic methanogens for hydrogen. Furthermore, dilute sulfuric acid pretreatment for delignification also significantly contributes to the low pH and ionic strength of thin stillage residue. 5-hydroxymethyl furfural and furfural are notorious fermentation inhibitors, and are also found in sugar cane stillage. The high ammonia, magnesium, and phosphate in the stillages can also promote the formation of struvite (magnesium ammonium phosphate hexahydrate) with higher pH values (>7) during anaerobic digestion which would require cleaning procedures as it can precipitate and foul the piping and reactor. On the other hand, with higher pH values, ammonia is present in its toxic unionized ammonia species which is then precipitated in harmless but fouling struvite. Unionized ammonia can cause hydrolytic and ammonolytic reactions leading to acid and amide formation [28]. This ammonia toxicity is less of an issue with low pH as with lower pH, formation of non-toxic ionized ammonium species is promoted.

Regardless of the above-mentioned characteristics of stillages that can be detrimental to anaerobic digestion, the COD of stillage derived from bioethanol production using energy cane and sugar cane was relatively high. The high COD indicates that there are high concentrations of organic matter available in thin stillage as substrate for anaerobic digestion. Mesophilic and thermophilic anaerobic digestion of the stillages were hence conducted in upflow anaerobic sludge blanket with packed-bed reactor technologies [9]. Bioreactor performance for both mesophilic (35 °C) and thermophilic (55 °C) hybrid bioreactors was very similar. Although, in general, thermophilic anaerobic digestion is assumed to produce methane more efficiently from the available carbon sources than mesophilic anaerobic digestion, microbial kinetics and other anaerobic digestion studies show similarity of thermophilic and mesophilic bioreactor performance [29, 30]. This implies that excessive heating of digesters may not be necessary, which possibly reduces operating costs. Optimal COD removal from cane stillage in the hybrid reactor system was reached at similar conditions for both mesophilic (35 °C) and thermophilic (55 °C) reactors and at a feed loading rate of 15–21 g COD/L/d. In this case, the pH ranged from 7.2 to 7.8, the amount of methane was up to 60–64 %, the specific methane production 0.2 L methane/g COD used and the COD removal up to 92–94 %. The high COD removal would result in effluent quality that falls within the permissible discharge limits for this parameter. Wastewater discharge in the Urbana & Champaign Sanitary District, for example, requires that the BOD (biological oxygen demand) of any grab sample does not exceed 2 g/L [31]. The BOD/COD ratio of thin stillage is 0.5 ± 0.1 (calculated from different types of thin stillage [22]). Therefore, the COD limit permitted for discharge was estimated as 4 g/L, higher than the COD content of the treated stillage. However, it is important to note that other water quality parameters particularly that of total nitrogenous content cannot be effectively reduced by anaerobic digestion. Hence, the anaerobic effluent cannot be directly released to environment but rather to a municipal sewerage for further treatment. Nevertheless, given the lower BOD and COD content in AD effluent that was recovered in the form of methane (i.e., energy source), the cost incurred on discharging this effluent to a municipal sewerage would be less than that incurred without any anaerobic digestion.

Although both types of stillages were successfully biodegraded in anaerobic digestion to form methane gas, there was a gap between our methane yield and COD removal. The COD balance discrepancy can be due to possible non-steady state conditions during certain phases of the reactor operation. Alternatively, the loss in the methane yield may be due to two factors, namely (i) dissolution of methane in the liquid fraction, (ii) competition between sulfate-reducing bacteria and methanogens for hydrogen, which, in turn, resulted in a reduction in methane yield. Sulfate-reducing bacteria (SRB) can also oxidize organic carbon in the absence of sulfate, which would contribute to COD reduction but not a corresponding increase in methane yield.

Also, differences in COD constituents of energy cane and sugar cane stillages were observed. For example, energy cane stillage was found in this study to have a high sugar content whereas sugar cane stillage was richer in volatile fatty acids (Table 1). Ethanol (158 mM) and glycerol were also present (10 mM) in sugar cane. Although both ethanol and glycerol were not assayed for energy cane stillage due to limited energy cane stillage sample availability, they are likely to be also present in both types of thin stillages and would constitute a good substrate for methane production [32]. Differences in the concentration of compounds between energy cane and sugar cane stillage may not only be due to the type or cultivar of the cane, but also to differences in pretreatment, enzymatic digestion and/or fermentation efficiency during bioethanol production that resulted from different test combinations at the demonstration plant.

Different COD constituents in each type of stillage and operating temperatures of reactors may have selected for distinct microbial populations. Microbial community dynamics based on differences in feedstock composition were studied but not the effect of operational strategy (mesophilic versus thermophilic, applied loading rate, hydraulic retention time or operation history) although the latter can likely also affect community dynamics. To illustrate, sugar cane stillage was more efficiently converted to methane and contained more acetic acid compared to energy cane stillage. This may indicate that there was more direct conversion of acetic acid to methane by acetoclastic methanogenesis in reactors treating sugar cane stillage. Accordingly, the *Methanosaeta*, a strictly acetoclastic methanogen [33], was more related to sugar cane stillage treatment under thermophilic conditions. Under mesophilic conditions, the genus *Methanosarcina* was abundantly present in reactors treating sugar cane stillage as well as in reactors treating energy cane stillage. The *Methanosarcina* genus comprises more versatile methanogenic species [34]. We speculate that this genus was more involved in acetoclastic methanogenesis when treating sugar cane stillage and in hydrogenotrophic methanogenesis in case of energy cane treatment. Mesophilic microorganisms that seem important for thin stillage degradation include species of the lactate-degrading Veillonellaceae, [35] as well as the Bacteroidales, Synergistaceae, Pedosphaerales, and *Kosmotoga.* Also, the sulfate-reducing Dethiosulfovibrionaceae and *Desulfovibrio* were more related to mesophilic conditions. This may indicate the greater importance of the reduction of oxidized sulfur compounds under these conditions, which can be due to the lower activation energy of such compounds compared to thermophilic conditions [36]. Based on past studies, furfural has been shown to be partially biodegraded by many bacteria including *Pseudomonas, Clostridium, Bacillus*, and *Desulfovibrio* species, and degradation of furfural to methane has been demonstrated by acclimated anaerobic sludge cultures [37, 38]. From our microbial characterization, bacteria able to biodegrade furfural and archaea that can further degrade metabolites obtained from furfural degradation to methane are present and may be able to assist in overcoming these notorious fermentation inhibitors.

In summary, this study provided baseline data on the characteristics of thin stillages, and demonstrated the feasibility of performing anaerobic digestion on these stillages to recover energy and to reduce the COD to a level that permits proper disposal. Future studies should aim to compare and evaluate the different reactor systems for the various operational conditions (e.g., applied organic loading rate and hydraulic retention time) so as to determine the optimal conditions to recover energy from this valuable resource.

## Conclusions

Thin stillage derived after the production of bioethanol with the use of energy cane and sugar cane as lignocellulosic biomass contains compounds, such as sugars and volatile fatty acids, that can be easily degraded to reduce the high amount of organic carbon present in thin stillage as well as for production of methane gas. Possible challenges for anaerobic digestion of thin stillage include low pH that may decrease anaerobic digestion and biogas production, high sulfate concentration that can reduce methane formation and struvite precipitation that may result in fouling the bioreactor and piping. The effluent obtained after digestion using high-rate hybrid reactors meets the standards for discharge in the municipal sewerage and can help to reduce the footprint of bioethanol production. Overall, energy cane and sugar cane stillage are feasible substrates for anaerobic digestion and promising sources for biofuel production in the form of methane gas.

## Additional file

10.1186/s13068-016-0532-z Three-way ANOVA significance analysis of hybrid bioreactor performance parameters; **Figure S1**. Methane percentage of total gas produced during anaerobic digestion of thin stillage; **Figure S2**. Specific methane production rate of hybrid reactors performing anaerobic digestion of thin stillage; **Figure S3**. Effluent pH of thin stillage fermenting hybrid reactors.

## Abbreviations

ANOVA: analysis of variance
BOD: biological oxygen demand
COD: chemical oxygen demand
CSTR: continuously stirred tank reactor
MHR: mesophilic hybrid reactor
OLR: organic loading rate
THR: thermophilic hybrid reactor
UASB: upflow anaerobic sludge blanket
VFA: volatile fatty acids
