# Supplementary material for: Lignocellulose-derived thin stillage composition and efficient biological treatment with a high-rate hybrid anaerobic bioreactor system
Source: Biotechnol Biofuels. 2016 Jun 6;9:120. doi: 10.1186/s13068-016-0532-z (PMC4895995; doi:10.1186/s13068-016-0532-z)

**Additional file 1**

**Table S1. Three-way ANOVA significance analysis of hybrid bioreactor performance parameters.** In a) specific methane production plus sCOD removal are shown and in b) effluent pH plus methane percentage of total gas. effint = effects and interactions of the different performance parameters, eff = only effects of the performance parameters, int = only interactions of the performance parameters, OLR = low compared to high organic loading rate, SL = energy cane thin stillage compared to sugar cane thin stillage, TEMP = mesophilic compared to thermophilic condition, Significance codes: *** = 0, ** = 0.001, * = 0.01, . = 0.05, ' ' = 1

a)

| **Variable** | **Test** | **Effect (symbol) /  interaction (symbols and colons)** | **p-value** | **Significance** |
| --- | --- | --- | --- | --- |
| **methane percentage of total gas** | effint | OLR | 0.17 |  |
|  |  | SL | 0.50 |  |
|  |  | TEMP | 0.21 |  |
|  |  | OLR:SL | 0.37 |  |
|  |  | OLR:TEMP | 0.47 |  |
|  |  | SL:TEMP | 0.29 |  |
|  |  | OLR:SL:TEMP | 0.40 |  |
|  | eff | OLR | 0.002 | ** |
|  |  | SL | 0.006 | ** |
|  |  | TEMP | 0.20 |  |
|  | int | OLR_high:SL | 0.13 |  |
|  |  | OLR_low:SL | 0.53 |  |
|  |  | OLR_high:TEMP | 0.41 |  |
|  |  | OLR_low:TEMP | 0.13 |  |
|  |  | SL:TEMP | 0.52 |  |
|  |  | OLR:SL:TEMP | 0.85 |  |

| **specific methane production** | effint | OLR | 0.04 | * |
| --- | --- | --- | --- | --- |
|  |  | SL | 0.76 |  |
|  |  | TEMP | 0.32 |  |
|  |  | OLR:SL | 0.03 | * |
|  |  | OLR:TEMP | 0.37 |  |
|  |  | SL:TEMP | 0.49 |  |
|  |  | OLR:SL:TEMP | 0.26 |  |
|  | eff | OLR | 0.41 |  |
|  |  | SL | 0.05 | * |
|  |  | TEMP | 0.09 | . |
|  | int | OLR_high:SL | 0.42 |  |
|  |  | OLR_low:SL | 0.20 |  |
|  |  | OLR_high:TEMP | 0.79 |  |
|  |  | OLR_low:TEMP | 0.12 |  |
|  |  | SL:TEMP | 0.97 |  |
|  |  | OLR:SL:TEMP | 0.65 |  |

b)

| **Variable** | **Test** | **Effect (symbol) /  interaction (symbols and colons)** | **p-value** | **Significance** |
| --- | --- | --- | --- | --- |
| **sCOD removal** | effint | OLR | 0.13 |  |
|  |  | SL | 0.53 |  |
|  |  | TEMP | 0.61 |  |
|  |  | OLR:SL | 0.28 |  |
|  |  | OLR:TEMP | 0.95 |  |
|  |  | SL:TEMP | 0.68 |  |
|  |  | OLR:SL:TEMP | 0.96 |  |
|  | eff | OLR | 0.004 | ** |
|  |  | SL | 0.004 | ** |
|  |  | TEMP | 0.59 |  |
|  | int | OLR_high:SL | 0.12 |  |
|  |  | OLR_low:SL | 0.48 |  |
|  |  | OLR_high:TEMP | 0.97 |  |
|  |  | OLR_low:TEMP | 0.04 | * |
|  |  | SL:TEMP | 0.93 |  |
|  |  | OLR:SL:TEMP | 0.14 |  |

| **effluent pH** | effint | OLR | 0.06 | . |
| --- | --- | --- | --- | --- |
|  |  | SL | 0.33 |  |
|  |  | TEMP | 0.39 |  |
|  |  | OLR:SL | 0.14 |  |
|  |  | OLR:TEMP | 0.17 |  |
|  |  | SL:TEMP | 0.32 |  |
|  |  | OLR:SL:TEMP | 0.32 |  |
|  | eff | OLR | 0.22 |  |
|  |  | SL | 0.64 |  |
|  |  | TEMP | 0.19 |  |
|  | int | OLR_high:SL | 0.99 |  |
|  |  | OLR_low:SL | 0.51 |  |
|  |  | OLR_high:TEMP | 0.86 |  |
|  |  | OLR_low:TEMP | 0.63 |  |
|  |  | SL:TEMP | 0.70 |  |
|  |  | OLR:SL:TEMP | 0.59 |  |

**Figure S1. Methane percentage of total gas produced during anaerobic digestion of thin stillage.** Effect of different organic loading rates of stillage derived from energy cane or sugar cane on hybrid reactor performance expressed as methane percentage of total gas under mesophilic and thermophilic conditions.

**
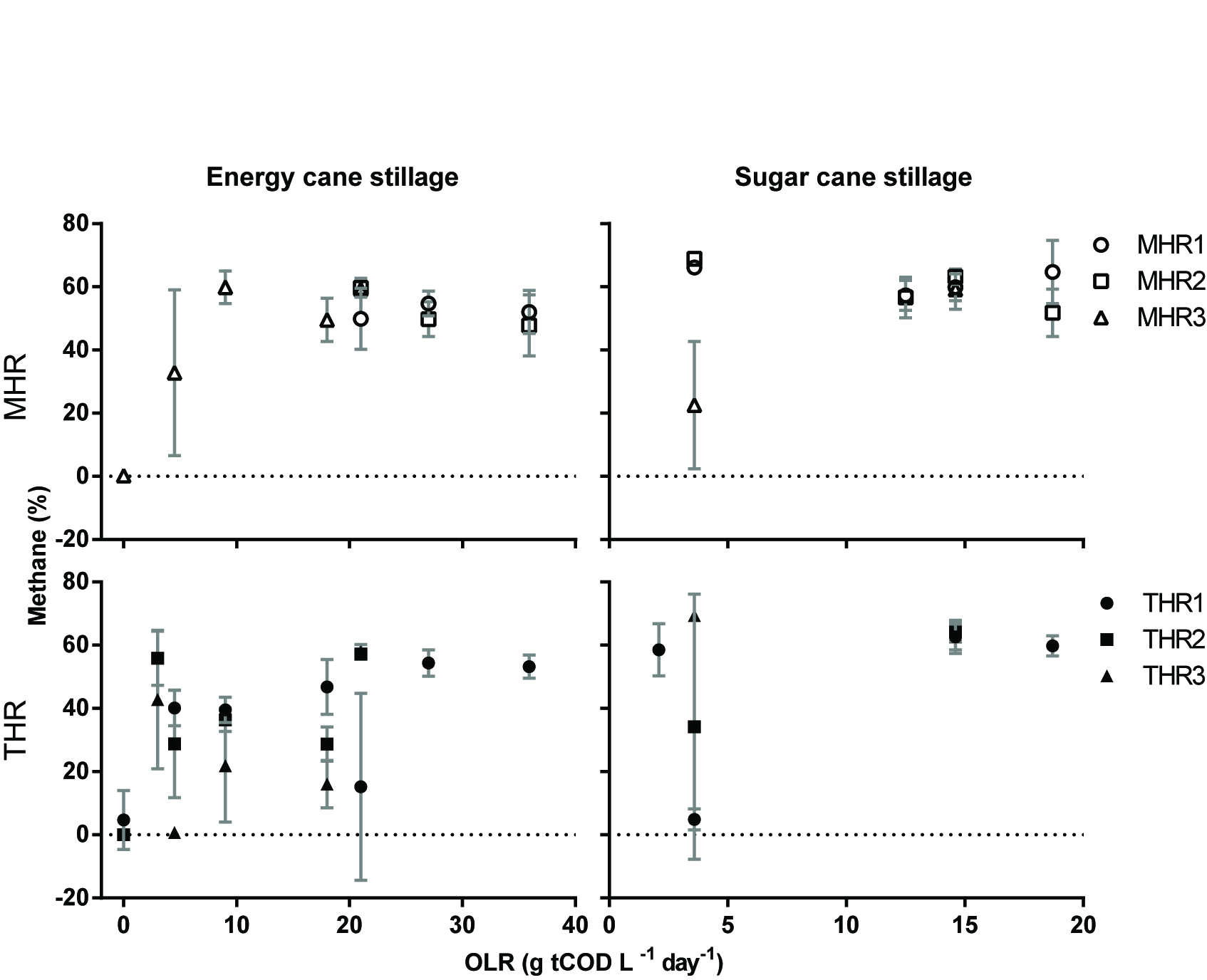
**

**Figure S2. Specific methane production rate of hybrid reactors performing anaerobic digestion of thin stillage.** Effect of different organic loading rates of stillage derived from energy cane or sugar cane on reactor performance expressed as specific methane production rate under mesophilic and thermophilic conditions.

**
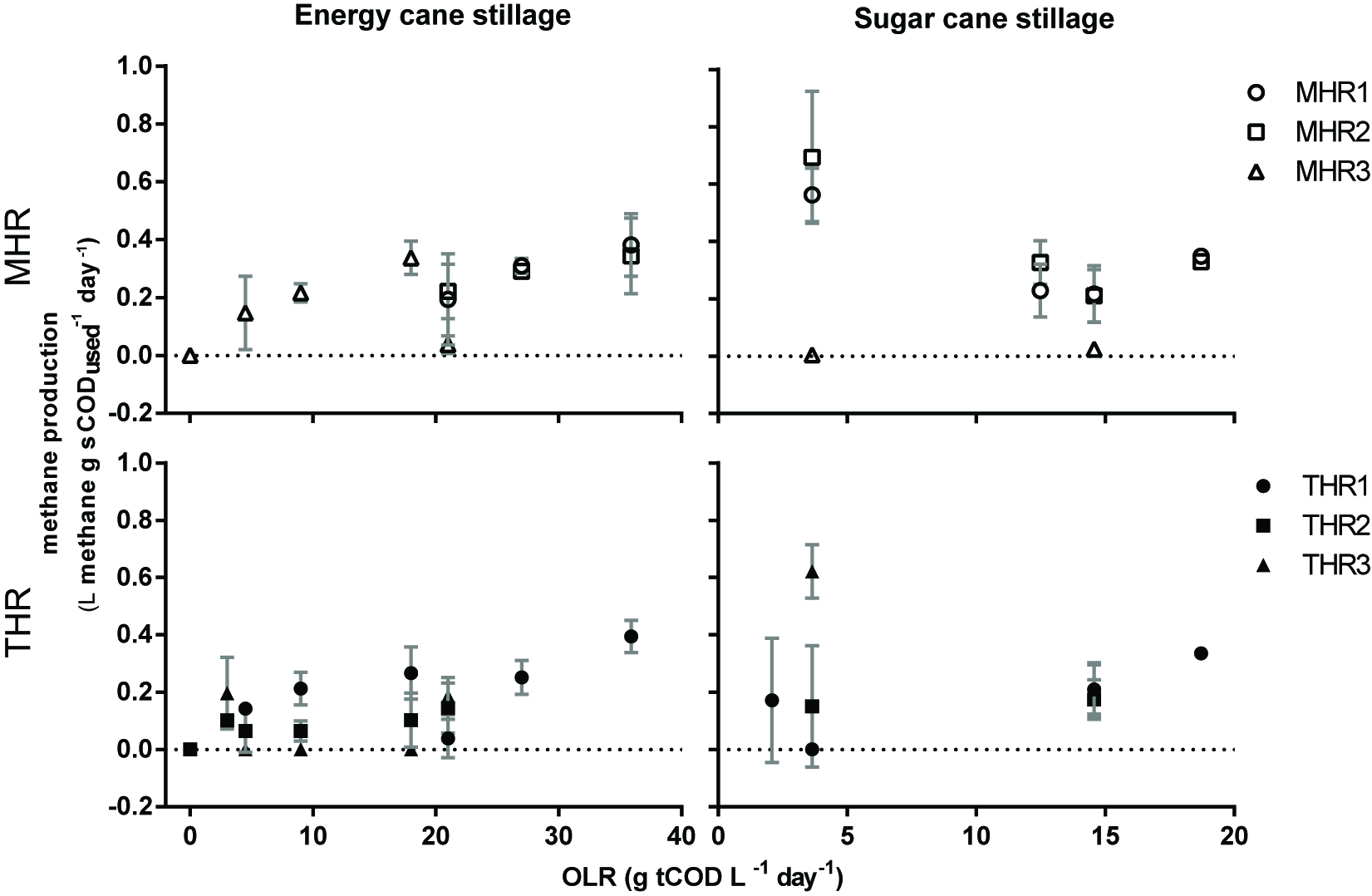
**

**Figure S3. Effluent pH of thin stillage fermenting hybrid reactors.** Effect of different organic loading rates of stillage derived from energy cane or sugar cane on reactor performance expressed as effluent pH under mesophilic and thermophilic conditions.


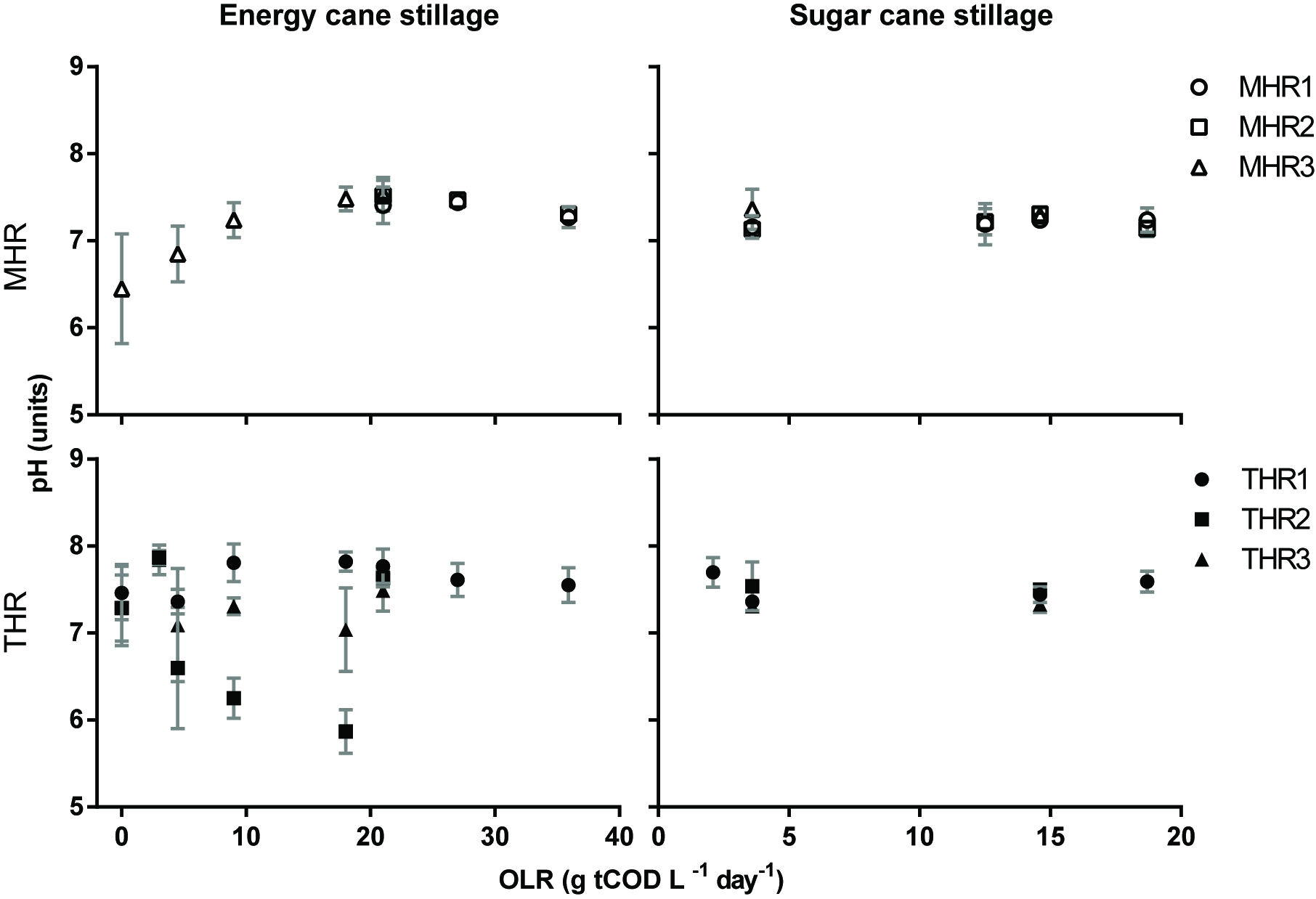

Supplement: Supplementary file 1 — 10.1186/s13068-016-0532-z Three-way ANOVA significance analysis of hybrid bioreactor performance parameters; Figure S1. Methane percentage of total gas produced during anaerobic digestion of thin stillage; Figure S2. Specific methane production rate of hybrid reactors performing anaerobic digestion of thin stillage; Figure S3. Effluent pH of thin stillage fermenting hybrid reactors. [file 13068_2016_532_MOESM1_ESM.docx]
